# Non-alcoholic fatty liver disease and associated risk factors in health care professionals in a community hospital in Brazil

**DOI:** 10.47626/1679-4435-2020-582

**Published:** 2021-03-03

**Authors:** Vitória dos Santos Magalhães, Thais Dall Acqua Jost, Henrique Mezzomo Pasqual, Anna Lourdes Gueller Becker, Luiza Maidana Marques, Mônica Manica, Bernardo Luis Lazzaroto Delani, João Pedro Langaro, Deise Terra Afonso, Lisia Hoppe, Ademir Orsolin, Ana Paula Pompeo Vartha

**Affiliations:** 1 Curso de Medicina, Universidade de Passo Fundo (UPF), Passo Fundo, RS, Brazil; 2 Curso de Medicina, Universidade Federal da Fronteira Sul, Passo Fundo, RS, Brazil; 3 Hospital São Vicente de Paulo, UPF, Passo Fundo, RS, Brazil; 4 Departamento de Hepatologia, UPF, Passo Fundo, RS, Brazil; 5 Clínica Medsom, Passo Fundo, RS, Brazil

**Keywords:** anxiety, stress, professionals, health, non-alcoholic fatty liver disease

## Abstract

**Introduction:**

Health care professionals are vulnerable to several health problems, including overweight, stress and anxiety. As such, non-alcoholic fatty liver disease is a likely diagnosis in this population.

**Objectives:**

To investigate the association between non-alcoholic fatty liver disease and levels of stress and anxiety in a sample of health care workers in a community hospital in the state of Rio Grande do Sul.

**Methods:**

The sample consisted of 107 health care workers who were interviewed and screened for non-alcoholic fatty liver disease based on clinical, imaging and laboratory parameters. Occupational stress was evaluated using Lipp’s Stress Symptom Inventory, and anxiety was assessed using the Hamilton Anxiety Rating Scale.

**Results:**

The mean age of the sample was 37.6 years. Most participants were female (89.1%) and the most frequent occupation was nursing technicians (83.2%). While 77.22% of participants did not report significant levels of stress, 30.7% did have mild anxiety. Statistical tests did not reveal a significant association between non-alcoholic fatty liver disease and stress (p = 0.688) or anxiety (p = 0.996).

**Conclusions:**

All participants with non-alcoholic fatty liver disease had some degree of anxiety, but only some experienced stress symptoms, according to Lipp’s Inventory. Statistical tests did not confirm an association between stress, anxiety and the presence non-alcoholic fatty liver disease. Nevertheless, the potential association between these variables should continue to be investigated given the global rise in the prevalence of non-alcoholic fatty liver disease and its implications for health care workers.

## INTRODUCTION

Work-related stress is a relevant occupational health issue in 21st century society. Health care workers are especially susceptible to high levels of anxiety and occupational stress due to excessive workloads, intense personal contact with inpatients, low wages, poor hospital infrastructure and an increasing patient load.

Occupational stress refers to the physical, mental and physiological responses that occur when job-related events overwhelm the individual’s coping skills, resulting in negative emotional reactions.^1^ Anxiety is organically expressed as a feeling of psychological discomfort, characterized by somatic manifestations such as cardiorespiratory symptoms, chest tightness and knots in the stomach or throat, as well as a state of chronic muscle contraction.^2^

The stressful situations experienced by health care workers have important consequences for the health of this population, leading to emotional symptoms such as anxiety, dissatisfaction and depression.^1^ Occupational stress can also have a negative impact on work life, decreasing the quality of care and interfering with the operation of health care services. As such, the negative effects of occupational stress extend far beyond the workers themselves, and ultimately influence patient care.

High levels of occupational stress increase susceptibility to illnesses such as hypertension, diabetes, obesity and liver disease.^3,4^ An example of one such illness is non-alcoholic fatty liver disease (NAFLD).

NAFLD is a clinical entity characterized by excess triglyceride accumulation in the liver, leading to histopathological features similar to those observed in alcoholic liver disease. However, it affects patients with a history of little to no alcohol consumption.^5-7^ Given the exposure of health care workers to several risk factors for NAFLD, it is important to investigate whether the presence of this condition is associated with stress and anxiety levels in this population.

## METHODS

This study was conducted at the Hospital São Vicente de Paulo (HSVP) in Passo Fundo, a city with a population of 196,739 inhabitants in the state of Rio Grande do Sul. It is considered the capital of the mid-highland region of the state, as its university hospital is a reference center for health care.^8^ The HSVP is a philanthropic institution that receives patients from several regions in the country. It has 700 beds, 504 (72%) of which are allocated to the Unified Health System (SUS).

Participants were recruited from the 1,501 health care professionals working at the HSVP. The sample was selected by cross-matching data from two previous studies, “Health care professionals’ health-related behaviors in National Health System clinics in the city of Pelotas-RS, Brazil”^9^ and “Global Epidemiology of nonalcoholic fatty liver disease - Meta-analytic assessment of prevalence, incidence and outcomes,”^10^ using the WinPepi software. Sample size was calculated assuming a prevalence rate of 11.8% for obesity in health care professionals. The prevalence of NAFLD in obese individuals was estimated to be 51% based on previous studies. Our calculations showed that a sample size of 90.3 individuals would be required to identify statistically significant (p < 0.05) associations between the variables of interest. To ensure a sufficient sample size, 107 participants were recruited.

All participants were directly involved in patient care. The sample was selected to reflect the occupational distribution of the total HSVP workforce. The final sample therefore included 5 nursing assistants (4.67%), 2 biologists (1.86%), 13 nurses (12.14%), one physical therapist (0.93%), 8 physicians (7.47%), one psychologist (0.93%), 75 nursing technicians (70.09%) and 2 radiology technicians (1.86%), for a total of sample size of 107 health care workers, all of whom worked at the HSVP from February to October 2017.

Inclusion criteria for the study consisted of actively working at the HSVP at the time of the study; a minimum age of 18; and written consent to participate in the study. The following exclusion criteria were applied: chronic liver disease (chronic hepatitis C, alcoholic liver disease, chronic hepatitis B, autoimmune hepatitis, primary sclerosing cholangitis, primary biliary cholangitis, hemochromatosis, Wilson’s disease); use of steatogenic medication (glucocorticosteroids, amiodarone, methotrexate, tamoxifen, valproic acid, antiretrovirals); alcohol intake over 30 g/day (men) or 20 g/day (women); current parenteral nutrition; lipodystrophy, hypobetalipoproteinemia, Reye syndrome, acute fatty liver of pregnancy, HELLP syndrome (hemolysis, elevated liver enzymes, low platelets); congenital metabolic disorders; and not giving written consent to participate in the study. After the initial interviews, 6 of the 107 patients were excluded, 4 due to hepatitis B and 2 due to excessive alcohol consumption.

As part of the study, all participants in the initial sample completed a questionnaire. Four participants were then excluded from the sample: one due to excessive alcohol consumption and 3 for having had hepatitis B. The remaining 103 participants underwent abdominal ultrasound examinations, all of which were conducted by a single experienced ultrasonographer using standardized equipment to quantify steatosis.^5^ NAFLD activity was graded based on the degree of steatosis, which was scored on a scale of 2 to 8. Hepatorenal contrast was used to assess the degree of steatosis and classify it as mild to moderate (score 2) or severe (score 3).

Patients with ultrasound evidence of NAFLD were then submitted to rapid hepatitis tests and laboratory examinations. At this stage, 2 additional patients were excluded from the study, one due to previous treatment for liver disease, and another for admitting to a higher alcohol intake than reported in the questionnaire. The remaining 101 patients underwent a complete blood count, platelet count, and measurements of serum creatinine; aspartate aminotransferase (AST) and alanine transaminase (ALT); bilirubin; alkaline phosphatase; gamma glutamyl-transferase; albumin; anti-HCV, HBsAg, anti-HBc, and anti-HBs antibodies; ferritin; transferrin saturation; ceruloplasmin; alpha-1-antitrypsin; anti-mitochondrial and anti-smooth muscle antibodies, in addition to the aforementioned rapid tests. These procedures were used to exclude other types of liver disease in order to make a diagnosis of NAFLD. NAFLD was diagnosed based on the presence of metabolic syndrome and altered lipid metabolism, regardless of transaminase levels.^11^ Examinations were conducted in a clinical analysis laboratory according to the standards of the Brazilian Society of Clinical Analysis (SBAC) and the National Program of Quality Control (PNCQ),^12^ and interpreted based on laboratory reference values.

Occupational stress was evaluated using Lipp’s Stress Symptom Inventory (LSSI). Participants were asked to rate their stress levels over the previous 24 hours, the previous week and the previous month. Results were classified into the following categories: alert (score > 7 in the past 24 hours), resistance (score > 4 in the previous week), exhaustion (score > 9 in the previous month) or no stress (if score ≤ 7, 4 or 9 in the past day, week and month, respectively).^13^

Anxiety was assessed using the Hamilton Anxiety Rating Scale (HAM-A). The instrument evaluates anxious mood, tension, fear, insomnia, cognitive difficulties, depressed mood, as well as motor, sensory, cardiovascular, respiratory, gastrointestinal, genitourinary and neurological symptoms. Items are rated on a 0 to 4 scale with the following response options: not present, mild, moderate, severe and very severe. These values are summed to yield a total score ranging from 0 to 56. These scores are then interpreted according to the following categories: a score of 17 or less indicates mild anxiety; a score of 18 to 24 suggests mild to moderate anxiety; scores of 25 to 30 indicate moderate to severe anxiety; and scores over 31 indicate extreme anxiety.^14^

## RESULTS

The initial sample comprised 107 health care workers employed by the HSVP. Four were excluded after the application of a questionnaire on risk factors for NAFLD and the diagnosis of other liver diseases.

The remaining 103 patients underwent an ultrasound examination, which revealed evidence of NAFLD in 27 (26.2%) cases, but no evidence of this condition in the other 76 (73.8%). One patient with findings consistent with NAFLD was later excluded after reporting a history of previous liver disease, leaving 26 patients (25.3%) in the NAFLD group. Another individual was also excluded from the sample of participants without NAFLD after reporting excessive alcohol use. The exclusions resulted in a final sample of 101 patients, 26 (25.7%) of whom presented with NAFLD. Steatosis was graded as mild in 14.8% of participants and moderate in 9.9% of cases, while 0.9% of the sample had focal steatosis. The remaining participants showed no evidence of liver disease.

The mean age of the sample was 37.37 (SD, 8.914) years, and participants ranged in age from 20 to 62 years. The sample was predominantly female, comprising 88 (87.1%) women and 13 (12.9%) men. A total of 82 participants were white (81.2%), while 15 (14.9%) were black and 4 (4%) were mixed-race.

The classification of body mass index (BMI) revealed that 2 participants (2%) were underweight, 46 (45.5%) had normal weight and 31 (30.7%) were overweight. Additionally, 19 (18.8%) patients had grade I obesity, 2 (2%) had grade II obesity and one (1.0%) had grade III obesity. The mean BMI in the sample was 25.91 and ranged from 18.00 to 41.90.

The classification of stress levels showed that 78 participants (77.2%) were in the “no stress” category, while 19 (18.8%) were in the “resistance” stage, three (3%) in the “exhaustion” and one (1%) in the “alert” stage.

Anxiety levels were mild in 31 cases (30.7%), mild to moderate in 28 (27.7%), moderate to severe in 26 (25.8%) and very severe in 16 cases (15.8%). A chi-square test did not reveal a significant difference in anxiety levels between patients with and without NAFLD (p = 0.0630) (Tables 1 and 2).

**Table 1:** Correlation between anxiety levels and presence of non-alcoholic fatty liver disease (NAFLD), Passo Fundo (RS), 2020 (n = 101)

Anxiety level	NAFLD	No NAFLD	Total
Mild			
Frequency	5	26	31
Percentage	16.1	83.9	100.0
Mild to moderate			
Frequency	9	19	28
Percentage	32.1	67.9	100.0
Moderate to severe			
Frequency	7	19	26
Percentage	26.9	73.1	100.0
Very severe			
Frequency	5	11	16
Percentage	31.3	68.8	100.0
Total			
Frequency	26	75	101
Percentage	25.7	74.3	100.0

**Table 2:** Correlation between stress levels and presence of non-alcoholic fatty liver disease (NAFLD), Passo Fundo (RS), 2020 (n = 101)

Stress level	NAFLD	No NAFLD	Total
No stress			
Frequency	20	58	78
Percentage	25.6	74.4	100.0
Alert			
Frequency	0	1	1
Percentage	0.0	100.0	100.0
Resistance			
Frequency	5	14	19
Percentage	26.3	73.7	100.0
Exhaustion			
Frequency	1	2	3
Percentage	33.3	66.7	100.0
Total			
Frequency	26	75	101
Percentage	25.7	74.3	100.0

**Table 3:** Correlation between anxiety, stress and non-alcoholic fatty liver disease (NAFLD), Passo Fundo (RS), 2020 (n = 101)

Spearman's rho	NAFLD grade	Anxiety level	Stress level
NAFLD grade			
Correlation coefficient	1.000	-0.092	-0.016
Sig. (two-tailed)	-	0.360	0.876
Anxiety level			
Correlation coefficient	-0.092	1.000	0.530^*^
Sig. (two-tailed)	0.360	-	0.000
Stress level			
Correlation coefficient	-0.016	0.530^*^	1.000
Sig. (two-tailed)	0.876	0.000	-

* Significant correlation at 0.01 (two-tailed).

The assessment of stress and anxiety levels in patients with different grades of NAFLD revealed no correlation between the grade of steatosis and levels of stress (p = 0.989) or anxiety (p = 0.630) (Tables 3 and 4).

**Table 4:** Correlation between higher levels of stress and non-alcoholic fatty liver disease (NAFLD), Passo Fundo (RS), 2020 (n = 101)

Stress level	NAFLD grade				
	Mild	Moderate	Focal	None	Total
No stress					
Frequency	12	7	1	58	78
Percentage	15.4	9.0	1.3	74.4	100.0
Alert					
Frequency	0	0	0	1	1
Percentage	0.0	0.0	0.0	100.0	100.0
Resistance					
Frequency	3	2	0	14	19
Percentage	15.8	10.5	0.0	73.7	100.0
Exhaustion					
Frequency	1	0	0	2	3
Percentage	33.3	0.0	0.0	66.7	100.0
Total					
Frequency	16	9	1	75	101
Percentage	15.8	8.9	1.0	74.3	100.0

## DISCUSSION

The researchers hypothesized that there would be a correlation between NAFLD and the levels of stress and anxiety experienced by health care workers. This was not supported by statistical analysis, as the present findings did not reveal a significant association between NAFLD and stress or anxiety levels. No prior studies appear to have analyzed the association between these variables, and as such, there is limited literature to which the present findings can be compared.

In Brazil, the population prevalence of hepatic steatosis and NAFLD has not been precisely determined, and few studies have looked into these illnesses or their prevalence in health care workers.^15^ However, the prevalence of NAFLD in the present study (25.7%) is consistent with worldwide prevalence estimates.^10^

Most participants in the present sample (n = 90, 89.1%) were female. Nursing and related occupations are traditionally held by women. Though men have been increasingly represented in these fields, most professionals in the area are still women. The mean age of professionals in this sample was 37.64 years, ranging from 20 to 63 years. These characteristics are similar to those of health care workers assessed in a previous study in a hospital in Santa Maria, a city in the state of Rio Grande do Sul, in which participants had a mean age of 41.3 years, and ranged in age from 24 to 69 years.^16^

In the present study, 84 (83.2%) participants were nurses or nursing technicians. Physicians were the third largest occupational group in the sample. This reflects the organization of health care work, where physicians often act as managers in larger teams of health professionals.^17^ These occupations therefore account for a significant percentage of hospital workers. As such, our data cannot determine whether the higher frequency of NAFLD in these occupations is due to a higher risk of disease in these workers or simply their numerical representation in the hospital workforce.

Both nurses and nursing technicians work long hours and have both day and night shifts. Data shows that night work can lead to increased BMI and metabolic syndrome. This is attributed to the interference of work on physiological processes such as the circadian rhythm, leading to changes in the secretion of melatonin, growth hormone, prolactin, leptin and glucocorticoids, leading to weight gain and the onset of metabolic disorders.^18,19^

The data collected in the present study also revealed that health care work may be associated with overweight, since the mean BMI in the sample was 26.02. Occupational factors can influence lifestyle, physical activity and eating habits. The occupational characteristics of health care work, such as adverse working conditions, long hours and excessive demands, have been found to be directly associated with excess weight.^20^ Additionally, both overweight and obesity lead to reduced quality of life and greater functional disability, since excess weight is closely related to a number of chronic illnesses.^18^

Occupational issues such as a lack of qualified personnel, medical supply shortages, excessive patient demands, direct contact with the families of critical care patients, complex workload and low wages may contribute to anxiety and stress. Additionally, health care workers often lack the time to pursue leisure activities, perpetuating a cycle of stress and anxiety.^3^

In the present study, anxiety was not significantly related to the presence of NAFLD. However, all patients with NAFLD reported some degree of anxiety, and as such, the association between these variables should continue to be examined. Most participants had mild-to-moderate or moderate-to-severe anxiety. Previous studies of health care workers have revealed associations between metabolic syndrome and anxiety in this population.^3^ Metabolic syndrome, in turn, is a major risk factor for NAFLD.^11^

The distribution of anxiety scores was similar across all categories of NAFLD severity. These findings suggest that severe NAFLD may not be related to higher levels of anxiety. However, this must also be further investigated, since few studies in the literature have examined the association between these variables.

Stress was also unrelated to the presence of NAFLD, since most (77.22%) participants with this condition did not report significant levels of stress. The correlation between stress levels and the degree of NAFLD was not significant (p = 0.98). The distribution of stress categories (no stress, alert, resistance and exhaustion) was also similar between individuals with and without NAFLD.

The lack of significant associations between these variables suggests that higher levels of stress may not be associated with higher grades of NAFLD. In fact, a significant portion of patients with mild and moderate NAFLD did not report significant levels of stress. Higher levels of stress (resistance and exhaustion), however, were associated with a lower frequency of high-grade NAFLD.

## CONCLUSION

The occurrence of NAFLD in hospital settings should be further studied, since health care professionals are exposed to several physical and psychological risk factors for this condition.

In the present study, most patients with NAFLD were nursing technicians. This was also the most common occupation in the sample. Future studies should examine this issue in greater detail to determine whether nursing technicians have a higher risk of NAFLD relative to other occupations, or if the higher number of cases in these workers is simply a consequence of their more frequent presence in hospitals. Our findings regarding BMI values in the sample also pointed to the need for interventions to encourage weight loss and the prevention of illnesses related to overweight and obesity, both of which are associated with reduced work-related functional capacity.

Though these findings were not statistically significant, there seems to be an association between the presence of anxiety and NAFLD, especially in individuals with mild-to-moderate and moderateto- severe anxiety. However, severe anxiety did not correlate with higher grade NAFLD. The presence of anxiety and NAFLD in health care workers should be examined by further studies. Stress was not significantly associated with the presence of NAFLD. Furthermore, there was no correlation between higher levels of stress and high grade NAFLD. Given the high prevalence of NAFLD, stress and anxiety in health care workers, and the scarcity of the literature on the topic, we emphasize the need for further studies on these issues.
